# The effect of a postbiotic produced by stabilized non-viable *Lactobacilli* on the health, growth performance, immunity, and gut status of colisepticaemic broiler chickens

**DOI:** 10.1007/s11250-022-03300-w

**Published:** 2022-09-09

**Authors:** Wafaa A. Abd El-Ghany, H. Fouad, R. Quesnell, L. Sakai

**Affiliations:** 1 Poultry Diseases Department, Faculty of Veterinary Medicine, Cairo University, Giza, 12211 Egypt; 2 Promovet Egypt Trade, Cairo, Egypt; 3 Transagra International Inc., Storm Lake, USA

**Keywords:** Chickens, *E. coli*, Growth performance, Immunity, Stabilized non-viable *Lactobacillus*

## Abstract

This work was designed to evaluate the efficacy of a postbiotic compound produced by stabilized non-viable *Lactobacilli* on the health, growth performance, immunity, and gut status against *Escherichia coli* (*E. coli*) challenge of broiler chickens. A total of 400, day-old broiler chicks were allocated into 4 equal groups (1–4) consisting of 100; each assigned into 2 equal replicates (50 each). Chickens in the 1st group were received the dry form of the compound at doses of 1 kg and 0.5 kg/ton feed for starter and grower, and the finisher diets, respectively. Chickens in the 2nd group were given the aqueous form of the compound in a dose of 4 mL/L of the drinking water during the first 3 days of life and at a day before and after each vaccination. Feed and water treatment regimens were administered to chickens in the 3rd group. Group 4 was kept without treatment. Each bird in the 1st, 2nd, 3rd, and 4th group was challenged with *E. coli* (O78) at 1-week-old. All groups were kept under observation till 5-week-old. Statistical analysis included one-way ANOVA and other methods as described with significant differences at *P* ≤ 0.05. The results indicated that feed and water treatments with the postbiotic compound induced more significant (*P* ≤ 0.05) amelioration of a disease picture, enhancement of growth performance, boosting of immune response, improvement of bursa of Fabricius/body weight ratio, and reduction of intestinal coliform count in challenged chickens when compared with challenged non-treated chickens. In conclusion, the postbiotic compound either in a dry and/or an aqueous form is recommended for improving the health, performance, and immunity of colisepticaemic broiler chickens.

## Introduction

The poultry industry is considered as one of the most important sources of income all over the world. Avian colibacillosis is an infectious disease that is caused by *Escherichia coli* (*E. coli*) (Lutful Kabir 2010). The disease is associated with heavy economic losses including mortality, decrease in productivity, and food-borne illnesses (Koutsianos et al. 2021). The most common avian pathogenic serogroups of *E. coli* are O78, O1, O2, and to some extent O15 and O55 (Ali et al. 2019). Antibiotic treatment is commonly used for the control of such infection. However, concern has been expressed that excessive use of antimicrobials, including those used at subclinical levels as growth promoters, is associated with the production of antibiotic-resistant strains of *E*. *coli* (Roth et al. 2019; Breijyeh et al. 2020; Radwan et al. 2021). These resistant strains can proliferate in the environment (Manyi-Loh et al. 2018) and infect humans either directly or via consumption of contaminated carcass (Valiakos and Kapna 2021). Mitigation strategies have been discussed (Ma et al. 2019), but successful strategies have been lacking. Thus, Food and Drug Administration Veterinary Feed Directive in 2017 prohibited the usage of antibiotics in farm animals as growth promotors (Veterinary Feed Directive 2019). Now, the urgent challenge of the poultry industry all over the world is to promote optimal production in parallel with providing safe product to the consumer. Some antibiotic alternatives have recently been used to improve poultry health while encouraging the production of food-borne disease-free products (Abd El-Ghany 2020).

Competitive exclusion compounds such as probiotics, prebiotics, and synbiotics are extensively used to prevent or reduce the intestinal colonization with *E. coli* especially in young broilers (Mohamed and Younis 2018). Although probiotics have been used to promote a healthy gut environment as well as growth performance and immune response of poultry, there is risk that they may acquire and transfer antibiotic resistance genes among microorganisms (Shazali et al. 2014). For instance, some probiotics of *Lactobacilli* species carry antibiotic-resistant genes for tetracycline and chloramphenicol that transfer among bacteria (Sharma et al. 2014). Besides, probiotics may have a negative influence on the host by increasing the severity of tissue inflammation (Tsilingiri et al. 2012). Additionally, many of these inoculants must be temperature controlled to maintain patency of live bacterial colonization. Subsequently, probiotics as living bacteria might not be used in the future. Recently, soluble non-viable probiotic metabolites or postbiotics have been used as a promising and potential substitute for both antibiotics and probiotics in poultry industry (Loh et al. 2010). Postbiotics are defined as secondary metabolites from probiotics without viable or living cells (Thanh et al. 2009). Postbiotics may be part of cell wall or cytoplasmic extracts of *Lactobacilli* species, stabilized bacteria, cellular products, or metabolic byproducts of fermentation (Johnson et al. 2019). They are not affected by acids, pH, or environmental conditions, so they can be kept safely, even under extreme temperature conditions, with longevity. Moreover, postbiotics of *Lactobacilli* origin have valuable components such as organic acids and bacteriocin which improve lactic acid bacteria growth (Loh et al. 2014). Components of cell wall and cytoplasmic extracts of numerous *Lactobacillus (L.)* species such as *L. acidophilus*, *L. plantarum*, *L. fermentum*, *L. casei*, *L. rhamnosus*, *L. paracasei*, *L. rhamnosus*, *L. delbrueckil* subsp. *Bulgaricus*, *L. gasseri*, *L. helveticus*, *L. reuteri*, and *L. johnsonni* were found to be highly effective postbiotics (Cicenia et al. 2016; Tiptiri-Kourpeti et al. 2016). Bacteria used for postbiotic production should be nonpathogenic, technologically suitable for industrial processes, acid and bile resistant, and good producers for antimicrobial substances that modulate immune responses and influence the metabolic activities of the gut (Dunne et al. 1999).

Therefore, the present study was planned to assess the efficacy of a postbiotic compound produced by *Lactobacilli* and including stabilized non-viable *Lactobacilli* on the health, growth performance, immunity, and gut status of challenged broiler chickens with *E. coli*.

## Materials and methods

The experiment was carried out in strict compliance with the recommendations of the National Regulations on Animal Welfare and following the guidelines approved by the Institutional Animal Care and Use Committee and all efforts were made to minimize suffering.

### Postbiotic compound

Stabilized non-viable *Lactobacilli* fermentation product (Culbac®, TransAgra’s International Inc., Storm Lake, Iowa, USA) is produced either in a dry or an aqueous form. The recommended manufacture dose of the dry form (Culbac® Animal Dry) is 1 kg/ton of the starter and grower diets and 0.5 kg/ton of the finisher diet. However, the dose of the aqueous form **(**Culbac® Animal Healthy Start) is 4 mL/L of the drinking water during the first 3 days of age and at 1 day before and after routine vaccination program.

### Chicks and experimental design

A total of 410, day-old broiler chicks (Hubbard breed) of mixed sex were obtained from a local hatchery in Giza governorate, Egypt. On arrival, ten birds were sacrificed and subjected for bacteriological culture examination on the selective media which proved absence of *E. coli* growth and the birds were free from *E. coli* infection. The remaining 400 chicks were allocated into 4 equal groups (1–4) consisting of 100; each assigned into 2 equal replicates (50 each). All the birds were kept in deep litter system under restricted hygienic measures for 5 weeks. Vaccination was done against Newcastle disease virus (NDv) using live HB1 and La Sota strains at 6- and 18-day-old, respectively, inactivated highly pathogenic avian influenza virus (HPAIv) H5N1 strain at 7-day-old, and infectious bursal disease virus (IBDv) using intermediate live strain at 13-day-old. All the vaccines were given via eye drop method, except influenza vaccine was given subcutaneously (S/C) at the back of the neck. Chickens were fed on commercial balanced diets containing local feed ingredients and formulated to meet the National Research Council requirements (NRC 1994) (Table 1). Birds were fed on starter, grower, and finisher diets at ages 1–15, 16–28, and 29–35 days, respectively. Feed and drinking water were provided ad libitum. The dry form of Culbac® was applied as feed treatment in groups 1 and 3, while the aqueous form was given in the drinking water for groups 2 and 3. Each chick in groups 1, 2, 3, and 4 was challenged S/C with 0.3 mL nutrient broth including 1 × 10^8^/mL of *E. coli* serotype (O78). Identified field strain of *E. coli* serotype (O78) was isolated from chickens with systemic colisepticaemia. The bacterial culture concentration was adjusted to 10^8^ colony forming unit (CFU) of *E. coli*/mL with previously defined nutrient broth (Fernandez et al. 2002).

**Table 1:** Composition of diet ingredients given for Hubbard broiler chickens during 5 weeks observation period

Composition	Starter	Grower	Finisher
Metabolized energy (kcal/kg)	3000	3150	3200
Crude protein %	23.0	22.0	19.0
Soybean meal (45%)	330.5	302.7	250.9
Yellow maize (9%)	57.94	57.94	57.94
Maize gluten meal (60%)	70.2	70.1	65.7
Fat	32	45	41
Lysine	2.9	2.7	3.5
Methionine	2.2	2.0	2.2
Dicalcium phosphate	18	18	18
Sodium chloride	4	4	4

Each gram of the mineral mixture of the diet contained: IU: vit. A 9000, vit. D3 2500, vit. E 17; mg, vit. K3 2.5, vit. B1 1.7, vit. B2 6.6, vit. B6 2.4, vit. B12 0.015; mg choline chloride 400, Mn 80, Fe 40, Zn 70, Cu 8, Se 0.3

### Measured parameters

#### Clinical observations

All groups were kept under complete observation for 5 weeks recording clinical signs, mortalities, and post-mortem lesions after *E. coli* challenge.

#### Growth performance

On arrival, random chicks were collected and the initial body weight was measured. The body weights, feed intake, and feed conversion ratio (FCR) were weekly assessed in all groups through the 5-week experimental period (Sainsbury 1984).

#### Immune response

Ten blood samples were collected from each group at 1-day-old and weekly until 5 weeks of age. The blood was kept in refrigerator for several hours and then centrifuged for serum separation. The antibody titers against NDv and HPAIv (H5N1) vaccines were estimated using haemagglutination inhibition (HI) test with 4 haemagglutinating units (HAU) (OIE 2002). Briefly, in each well of HI plates, 4 HAU of virus/antigen were added, kept for 30 min, and then 0.050 mL of 0.5% chicken red blood cells was added for 30 min. Serum samples including positive and negative controls were distributed in wells. Inhibition at a dilution of 1/16 (4 log_2_ when expressed as the reciprocal) or more against 4 HAU was considered as positive HI. Moreover, the antibody titers against IBDv were measured at the 2nd, 3rd, 4th, and 5th week of age using enzyme-linked immunosorbent assay (ELISA) test (Snyder et al. 1984).

#### Bursa/body weight ratio

On weekly basis, 5 randomly selected chickens from each group were sacrificed, weighed, and the bursae of Fabricius were incised and weighed to determine bursa/body weight ratio.

#### Total coliform count

Five birds from each group were sacrificed at 5 weeks old. One hundred grams of the intestinal contents from different parts of the intestine (duodenum, ileum, and caecum) were homogenized with 0.5 mL of sterile buffered peptone, and tenfold serial dilutions for each homogenate from the initial dilution (10^−1^) were made. Approximately 0.1 mL of each dilution was incubated on a MacConkey agar plate at 37 °C for 24 h. The results of the count were expressed as the number of the organism log_10_ CFU/g of the intestinal content (Huang et al. 2019).

#### Histopathological examination

At 5-week-old, 5 birds from each group were sacrificed. Specimens from liver and intestine were collected, fixed in 10% neutral buffered formalin, dehydrated in different grades of ethyl alcohol, and embedded in paraffin wax. Microtetomy of the tissue to 5 µm thickness was done and stained with hematoxylin and eosin stain for histopathological examination by the light microscope (Bancroft and Gamble 2007).

### Statistical analysis

The results were statistically analyzed according to Snedecor and Corchran (1980). One-way ANOVA was adopted using SAS® software general liner models procedure. Significant differences between treatment means were considered significant at level *P* ≤ 0.05.

## Results

### Clinical observations

Signs of depression, off food, ruffling, congestion of mucous membrane of the buccal cavity, conjunctiva, and comb and wattle, increasing the consumption of water, and greenish diarrhea were observed 2 days post *E. coli* challenge. The signs picture was absent in the treated groups as compared to the non-treated group. The effects of different postbiotic treatments on the mortality and protection rates of *E. coli*-challenged chickens are presented in Table 2. Chicken’s mortality began 3 days post-challenge and continued for 4 days in non-treated birds, while it subsided in the treated chickens 2 days post-challenge. Combined feed and water treatments with the postbiotic compound showed the highest protection rate of 93%, whereas the non-treated challenged group revealed protection rate of 73%. The protection rates of postbiotic treatment in feed and water were 90% and 88%, respectively. The post-mortem lesions of *E. coli*-challenged chickens were septicemia, hemorrhages on the internal organs, liver congestion and necrosis, and enteritis. However, this lesions picture was not seen in postbiotic-treated chickens.

**Table 2:** Effect of postbiotic treatments on clinical status in different groups

Group	Treatment	*E. coli*	Mortality %	Protection %
1	Feed	+	10	90
2	Water	+	12	88
3	Feed and water	+	7	93
4	-	+	27	73

Number of birds/group (*n* = 100)

Mortality % = number of dead birds/total number of birds (*n* = 100)

Protection % = number of survived birds/total number of birds (*n* = 100)

### Growth performance

The results of the growth performance parameters of different groups are shown in Table 3. Application of postbiotic compound in feed and water significantly (*P* ≤ 0.05) improved the performance parameters of broilers. The highest significant average body weights were recorded in chickens treated with combined postbiotic treatments in feed and water (1360.7 g), followed by feed (1245.9 g) and water (1196.5 g) treatments when compared with non-treated chickens (987.8 g). The FCR (1.6) was recorded in all the postbiotic-treated groups which was better than (2.2) of the non-treated group.

**Table 3:** Effect of postbiotic treatments on performance parameters in different groups

Group	Treatment	*E. coli*	Average body weight/g (mean ± SD)/age/week					FCR
			1st	2nd	3rd	4th	5th	
1	Feed	+	140.1 ± 7.6^a^	290.3 ± 6.9^b^	578.9 ± 22.6^b^	890.6 ± 65.3^a^	1245.9 ± 65.0^a^	1.61^b^
2	Water	+	138.9 ± 3.2^a^	270.9 ± 8.1^ab^	556.1 ± 25.4^ab^	863.9 ± 54.7^b^	1196.5 ± 46.8^b^	1.63^b^
3	Feed and water	+	142.5 ± 5.4^a^	320.6 ± 5.7^a^	607.5 ± 27.1^a^	910.9 ± 49.6^a^	1360.7 ± 40.2^a^	1.61^b^
4	-	+	135.1 ± 3.1^a^	299.9 ± 7.9^b^	530.6 ± 19.8^ab^	815.7 ± 54.8^b^	987.8 ± 70.1^c^	2.2 ^a^

Means with different letters (a, b, c) within the same column are significantly different at *P* ≤ 0.05

*FCR*, feed conversion ratio

### Immune response

The results of immune response against NDv vaccine are showed in Table 4. The HI test results revealed that the geometric mean maternal antibody titer of day-old-chicks was 6.8. The HI titers against NDv vaccine were significantly (*P* ≤ 0.05) the highest in chickens treated with mixed feed and water postbiotic treatment along 5 weeks experimental period when compared single water or feed postbiotic treatment as well as the non-treated group. Moreover, the lowest significant (*P* ≤ 0.05) means of HI titers were recorded in the non-treated group in comparison with the treated groups.

**Table 4:** Effect of postbiotic treatments on haemagglutination inhibition titers to Newcastle disease virus vaccine in different groups

Group	Treatment	*E. coli*	HI titers (mean ± SD) (log_2_)/age/week				
			1st	2nd	3rd	4th	5th
1	Feed	+	7.71 ± 0.2^a^	7.56 ± 0.2^a^	7.30 ± 0.1^ab^	7.06 ± 0.1^ab^	6.75 ± 0.1^ab^
2	Water	+	7.37 ± 0.1^b^	7.20 ± 0.1^ab^	7.07 ± 0.2^ab^	6.79 ± 0.3^b^	6.28 ± 0.1^b^
3	Feed and water	+	7.97 ± 0.2^a^	7.80 ± 0.1^a^	7.76 ± 0.1^a^	7.21 ± 0.1^a^	6.96 ± 0.2^a^
4	-	+	6.88 ± 0.1^b^	6.98 ± 0.3^b^	6.79 ± 0.1^b^	6.22 ± 0.2^b^	5.89 ± 0.1^b^

Means with different letters (a, b, c) within the same column are significantly different at *P* ≤ 0.05

Haemagglutination inhibition: (HI)

Regarding the humoral immunity against HPAIv (H5N1) vaccine, the results are presented in Table 5. The humoral immunity against HPAIv (H5N1) vaccine showed significant (*P* ≤ 0.05) difference between postbiotic-treated and non-treated chickens. However, combined feed and water treatment of postbiotic gave the best immune response along the whole experiment.

**Table 5:** Effect of postbiotic treatments on haemagglutination inhibition titers to HPAI (H5N1) in different groups

Group	Treatment	*E. coli*	HI titers (mean ± SD) (log_2_)/age/week				
			1st	2nd	3rd	4th	5th
1	Feed	+	8.21 ± 0.57 ^ab^	8.65 ± 0.20^ab^	9.66 ± 0.33^b^	8.78 ± 0.16^ab^	8.95 ± 0.05^ab^
2	Water	+	8.53 ± 0.41^ab^	8.94 ± 0.25^ab^	8.33 ± 0.71^ab^	9.65 ± 0.30^b^	9.90 ± 0.52^a^
3	Feed and water	+	8.61 ± 0.24^ab^	8.97 ± 0.39^ab^	9.79 ± 0.74^b^	10.35 ± 0.16^a^	10.12 ± 0.34^a^
4	-	+	8.03 ± 0.23^bc^	7.81 ± 0.54^bc^	7.18 ± 0.30^c^	6.89 ± 0.11^c^	7.06 ± 0.41^c^

Means with different letters (a, b, c) within the same column are significantly different at *P* ≤ 0.05

Haemagglutination inhibition: (HI)

**Fig. 1:**
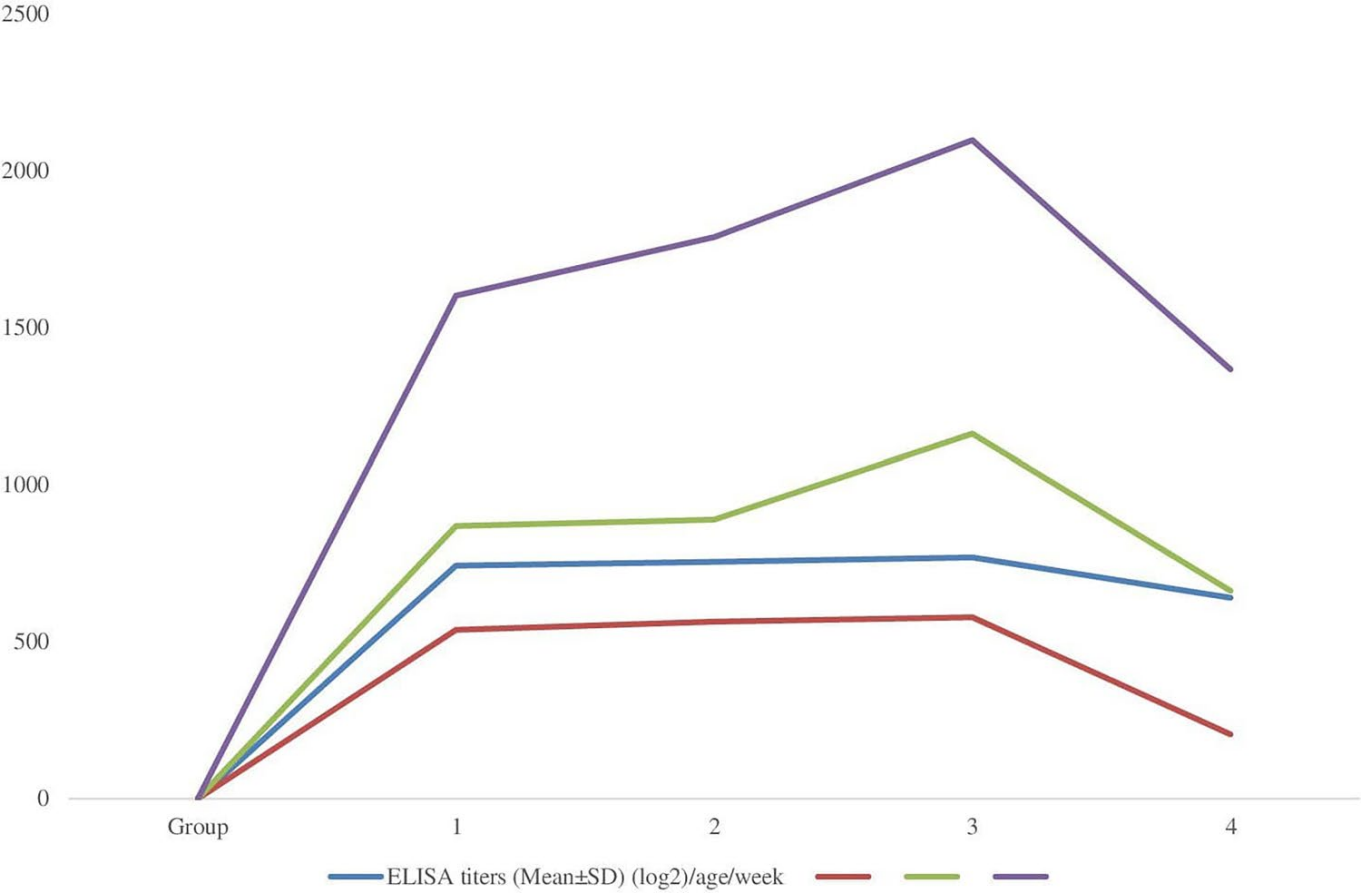
Effect of postbiotic treatments on the humoral immune response to IBDv using ELISA test in different groups

The humoral immune response to IBDv was measured from the 2nd till the 5th week of life and the mean ELISA titers showed significant (*P* ≤ 0.05) increase in all the postbiotic-treated groups when compared with the challenged non-treated group (Fig. 1). However, chickens given a postbiotic in feed and water showed the best means from the 2nd till the 5th week of age.

### Bursa/body weight ratio

The measured mean bursae of Fabricius/body weight ratio in Table 6 indicated that the highest significant (*P* ≤ 0.05) ratios were in the postbiotic-treated groups when compared with the non-treated group. At the end of the study, the mean bursae/body weight ratios were 0.291, 0.288, and 0.272 in combined feed and water, feed, and water in the postbiotic-treated groups, respectively; however, this ratio was 0.203 in the non-treated group.

**Table 6:** Effect of postbiotic treatments on bursa of Fabricius/body weight ratio in different groups

Group	Treatment	*E. coli*	Bursa/body weight ratio (g) (mean ± SD)/age/week				
			1st	2nd	3rd	4th	5th
1	Feed	+	0.086 ± 0.02^ab^	0.192 ± 0.05^a^	0.199 ± 0.01^ab^	0.226 ± 0.07^ab^	0.288 ± 0.03^a^
2	Water	+	0.080 ± 0.06^ab^	0.156 ± 0.01^ab^	0.194 ± 0.07^ab^	0.220 ± 0.08^ab^	0.272 ± 0.07^ab^
3	Feed and water	+	0.099 ± 0.05^a^	0.194 ± 0.08^a^	0.228 ± 0.02^a^	0.253 ± 0.06^a^	0.291 ± 0.09^a^
4	-	+	0.069 ± 0.09^b^	0.135 ± 0.07^b^	0.151 ± 0.03^b^	0.193 ± 0.01^b^	0.203 ± 0.05^b^

Means with different letters (a, b, c) within the same column are significantly different at *P* ≤ 0.05

Bursa/body weight ratio = [Bursa weight (g) / body weight (g)] × 100

### Total bacterial count

In comparison with non-treated challenged chickens, postbiotic-treated chickens showed significant (*P* ≤ 0.05) reduction in the total intestinal coliform count at the end of observation period (Table 7). The intestinal coliform counts were 3.98 × 10^5^, 4.72 × 10^5^, and 4.10 × 10^5^ in combined feed and water treatment, water treatment, and feed treatment, respectively. Meanwhile, the total coliform count in non-treated chickens was 8.06 × 10^6^.

**Table 7:** Effect of postbiotic treatments on intestinal coliform count in different groups

Group	Treatment	*E. coli*	Intestinal coliform count log_10_ (CFU/g)
1	Feed	+	4.10 × 10^5^ ± 0.01^a^
2	Water	+	4.72 × 10^5^ ± 0.03^a^
3	Feed and water	+	3.98 × 10^5^ ± 0.02^a^
4	-	+	8.06 × 10^6^ ± 0.03^b^

Means with different letters (a, b, c) within the same column are significantly different at *P* ≤ 0.05

### Histopathological examination

**Fig. 2:**
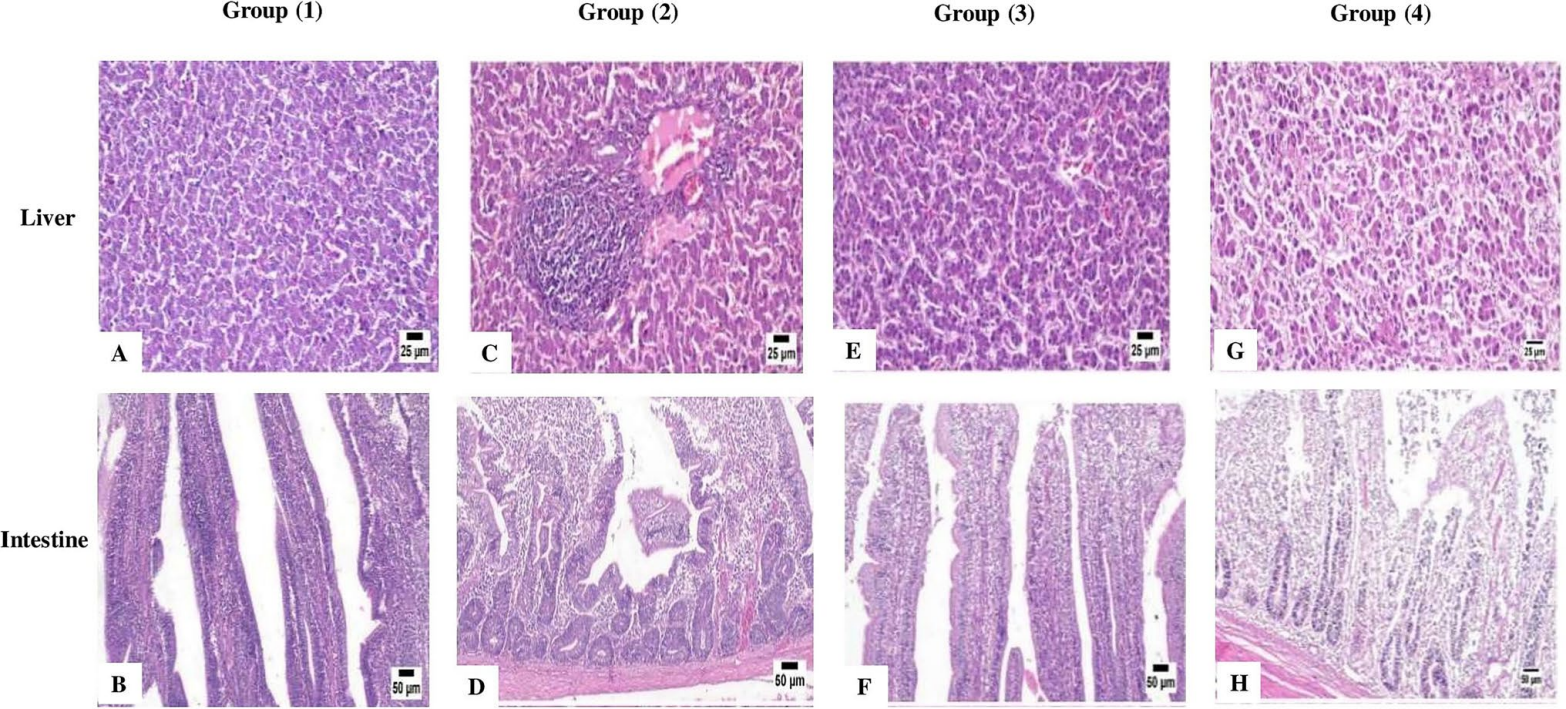
The results of the histopathological examination of liver and intestine in different groups

Figure 2 shows the results of the histopathological examination of liver and intestine in different groups. In group treated with the postbiotic in feed, the liver showed apparently normal hepatic parenchyma (A), while the intestine showed mild inflammation (B). Postbiotic treatment in water revealed focal portal hepatitis (C) and moderate enteritis (D). However, in a group that received combined feed and water treatments, apparently normal hepatic parenchyma (E) and mild enteritis (F) were observed. The *E. coli*-challenged group with *E. coli* that received no treatment exhibited disorganization of the hepatic plates admixed with numerous locations of scattered sporadic cell necrosis (G) and severe necrosis of the intestinal villi with accumulation of inflammatory cells and tissue debris in the submucosa (H).

## Discussion

The devastating effects of avian colibacillosis are particularly prevalent in the poultry field, and poor biosecurity and husbandry practices, especially in developing countries, create a difficult paradigm for improvement without some easily applied intervention strategies (Ebrahimi-Nik et al. 2018). The disease induces significant economic annual losses in the global poultry industry (Roth et al. 2019). The resulting emergence and rapid dissemination of antibiotic-resistant *E. coli*, believed to be a result of low levels of antibiotics in feed, have resulted in a reduced efficacy of specific antibiotic classes and thus might pose a substantial risk in humans’ health (Belanger et al. 2011). Accordingly, it was necessary to find viable alternative resources to protect production levels and still maintain the health of the birds (Seal et al. 2013). One of these substitutes is “biotic feed additives” such as probiotics, prebiotics, and postbiotics (Klemashevich et al. 2014). The compound used in this study (Culbac®) is produced by fermentation process of proprietary strain of *Lactobacilli* and then a unique, proprietary method for protecting surface proteins and stabilization method for rendering the bacteria non-viable or dead. The non-viable bacterial fermentation product (postbiotic) is exposed to extensive processing to modify the cell contents and allow exposure of the cell wall to the digestive bacteria.

There was a notable decrease in the severity of the *E. coli* clinical observations after feed and water postbiotic treatments. Similarly, previous studies indicated postbiotic metabolites of specific strains of *Pediococcus acidilactici*, *Enterococcus faecium*, *L. reuteri*, and *L. acidophilus* reduced mortality and lesion scores of *C. perfringens*-challenged broiler chickens as compared to challenged non-treated chickens (Johnson et al. 2019). Moreover, the postbiotic from specific *L. plantarum* strains showed a trend of lower mortality in broiler chickens as compared with other treatments such as antibiotic growth promotor and ascorbic acid (Humam et al. 2019). These studies, in conjunction with the current study, indicate options for productive antibiotic-free production of poultry globally.

In this study, postbiotic treatment of broiler chickens improved the weight gain and the FCR while minimizing effects from exposure to pathogenic *E. coli* (078). It has been documented that postbiotics can be used as feed additives to promote the health and growth performance in broilers (Kareem et al. 2017) and layers (Choe et al. 2012; Loh et al. 2014). Thanh et al. (2009) reported that chickens fed combinations of metabolites produced by *L. plantarum* had higher final body weight and weight gain compared with those fed the negative control diet. Heat-stressed broiler chickens fed on a diet containing postbiotics of *L. plantarum* showed elevated weight gain and superior feed conversion efficiency as a result of increasing hepatic insulin-like growth factor 1 mRNA expression level (Humam et al. 2019). In addition, a combined mixture of postbiotic and prebiotic in the diet of broiler chickens enhanced the total body weight and the feed efficiency in conjunction with increasing liver insulin like growth factor 1 and growth hormone receptor mRNA expressions (Kareem et al. 2016). Improvement in weight gain was observed after treatment of *C. perfringens*-challenged broiler chickens with a postbiotic when compared with non-treated control (Johnson et al. 2019). However, Rosyidah et al. (2011) found no significant difference in body weight or weight gain of chickens fed on metabolites and combination of metabolite and acidifier and those fed positive and negative control diets. It is likely to mention that Hubbard broilers that used in this study have an excellent FCR and robustness which may support the action of the tested postbiotic.

These findings may be a result of the ability of postbiotics to reduce the number of pathogenic intestinal microorganisms, leading to better gut health and growth performance. Postbiotics may be also similar to probiotics in regard to the enhancement of nutrient transporter gene expression (Na + -dependent glucose, galactose transporter, and long-chain acyl CoA dehydrogenase genes) which promote broiler growth performance (Jahromi et al. 2016). Additionally, *Lactobacilli* species may increase the utilization of nutrients due to upregulation of nutrient gene expression resulting in improvement of the bodyweight of broilers (Kalavathy et al. 2003). Organic acids and bacteriocins are antimicrobial metabolites of postbiotics that may decrease the pH and prevent the proliferation of pathogens in the gut of animals (Aguilar-Toalá et al. 2018). Moreover, supplementation with postbiotics could enhance growth performance and health via improvement of physiological parameters including immune status, gut health, intestinal villus, increasing lactic acid bacteria, and decreasing *Enterobacteriaceae* and intestinal pH (Loh et al. 2010; Kareem et al. 2016).

The immune response against NDv, HPAIv, and IBDv vaccines was modulated after postbiotic treatments in the present study. Moreover, improved bursa of Fabricius/body weight ratios were observed in postbiotic-treated chickens as compared with non-treated chickens. It is known that *E. coli* infection is an immunosuppressive pathogen of poultry (McGruder and Moore 1998) as this pathogen can damage the immune system of chickens in terms of lymphocyte depletion in both bursa and thymus tissues (Nakamura et al. 1990). Hegazy et al. (2010) found that infection of chickens with *E. coli* prior to vaccination with IBDv vaccine resulted in more decrease in ELISA antibody titers in comparison with vaccinated non-infected chickens. The current results agree with those of Johnson et al. (2019) who found that postbiotic metabolites mixture of *Pediococcus acidilactici*, *L. reuteri*, *Enterococcus faecium*, and *L. acidophilus* was able to stimulate the immune response in *C. perfringens* infected broiler chickens. Moreover, the levels of immunoglobulin M (IgM) and IgG were significantly higher in broilers that received postbiotics in feed than those that received ascorbic acid. Stimulation of immune response in birds after postbiotic treatment might be related to the presence of peptidoglycan (β-glucan) in 90% of the dry weight of *Lactobacilli* with lipopolysaccharides and teichoic and lipoteichoic acids in the bacterial cell wall (Adams 2010). In addition, after *Lactobacilli* lysis or degradation by host gastric acid, the bacterial DNA (CpG motifs) released and recognized by the host as a foreign antigen has been shown to stimulate both the cell-mediated and humoral immune responses (Kant et al. 2014).

The current results showed that the total intestinal coliform count was reduced in the postbiotic-treated groups while it was high in the non-treated group. The bacteriostatic and bactericidal activities of postbiotics have been reported. During fermentation of *Lactobacilli* bacteria, metabolites are produced, released, and stimulated the growth of beneficial microorganisms (Caldwell 2016). Some of beneficial bacteria are fastidious and require a lot of nutrients such as amino acids, energy (sugars), and metabolites (vitamin B complex) provided by the host. Accordingly, postbiotics can provide these bacteria by nutrients such as inulin or cellulose that present in their cell walls. In addition, postbiotics can reduce the multiplication of harmful bacterial in the gut. Parallel results were found in pigs which showed a reduction in *E. coli* count after treatment with cell free extracts of *Lactobacilli* fermentation process (Pollman et al. 1982; Blomberg et al. 1993; Thu et al. 2011). A postbiotic metabolite of *L. plantarum* either alone or in combination with a prebiotic showed inhibitory effects of some pathogens such as *E. coli*, *Salmonella typhimurium*, vancomycin-resistant *enterococci*, and *Listeria monocytogens* (Thanh et al. 2009; Van Thu et al. 2011; Choe et al. 2013; Kareem et al. 2014). A significant increase in lactic acid bacteria and decrease in *Enterobacteriaceae* count were observed in broiler chickens fed on *L. plantarum* (Rosyidah et al. 2011) or a mixture of postbiotic and inulin (Kareem et al. 2016). Furthermore, the postbiotic produced from a mixture of *L. plantarum* strains significantly increased the total bacteria and *Lactobacilli* counts but decreased *Salmonella*, *E. coli*, and *Enterobacteriaceae* counts in broilers when compared to the control groups (Humam et al. 2019). Studies in humans showed that lipoteichoic acids of *L. acidophilus* and *L. johnsonni* supported human gut homeostasis and treated diseases caused by Gram-negative bacteria (Vidal et al. 2002). Also, *L. paracasei* cells supernatant protected tissues from invasive *Salmonella* (Tsilingiri et al. 2012).

The histopathologic examinations in this study revealed that the *E. coli*-challenged group exhibited disorganization and necrosis of the hepatic cells plus severe necrosis of the intestinal villi with inflammatory cells. The current observation is in tandem with that of Abalaka et al. (2017). In contrast, chickens treated with combined postbiotic treatment in feed and water or in feed alone showed apparently normal hepatic parenchyma and mild intestinal inflammation. Previous studies have shown that improvement of intestinal villi height was observed in broiler chickens treated with metabolite combinations in feed (Thanh et al. 2009; Loh et al. 2010). Moreover, addition of postbiotics and inulin combinations to the diet of broiler chickens beneficially altered the intestinal mucosal architecture in terms of longer villi (Kareem et al. 2016). Recently, Humam et al. (2019) demonstrated that a mixture of *L. plantarum* metabolites significantly increased the intestinal villus height to crypt depth ratio. Positive modification of liver architecture after postbiotic treatment may be attributed to the ability of *Lactobacillus* species to alleviate the severe damage of the liver by increasing the antioxidant enzyme activities in the blood (Chen et al. 2022). These findings provide new hope for growing healthy, productive animals while minimizing use of traditional antibiotics in production operations.

The reviewed postbiotic compound utilized in this study has not been previously tested in such a virulent challenge model, but neither have probiotics, phytobiotics, nor synbiotics been compared in a challenge model with *E. coli* that is demonstrated to produce colisepticaemia in broilers. Defining new opportunities for growth support in poultry while not inducing additional potential pressures for antibiotic-driven antibacterial-resistance mechanism proliferation, nor probiotic proliferation of resistance mechanisms, would be a great asset for poultry production globally. This study demonstrates an early inroad to the use of postbiotics as a valuable tool for the poultry industry (Fancher et al. 2020).

In conclusion, this study showed that the used postbiotic (Culbac®) either in a dry or an aqueous form was effective in amelioration of colisepticaemia in broiler chickens. Moreover, the synergistic beneficial action of both treatment regimens when supplemented together was evident. Combined feed and water treatment with the postbiotic was even better than a separate treatment. Under the present experimental conditions, reduction of the clinical disease picture, improvement of performance parameters, modulation of the humoral immune response to NDv, HPAIv, and IBDv vaccines, and decreasing in intestinal coliform count were proven after postbiotic treatment of *E. coli*-challenged chickens. This may indicate the growth promoting, immunomodulatory, and protective activities against pathogenic bacteria with the tested natural compound (Culbac®) which may be used in the near future for opposing this serious devastating infections in poultry. With the current climate of “no antibiotics ever,” the poultry industry is looking for options, and this work provides a new option for producers.

## Data Availability

The datasets generated during the current study are available from the corresponding author on reasonable request.
